# Supplementary material for: Estimating the frequency of multiplets in single-cell RNA sequencing from cell-mixing experiments
Source: PeerJ. 2018 Sep 3;6:e5578. doi: 10.7717/peerj.5578 (PMC6126471; doi:10.7717/peerj.5578)
Supplement: Supplemental Information 4 — This file contains an HTML rendering of the Jupyter notebook in Supplemental file 3. [file peerj-06-5578-s004.html]

calcmultiplet\_R


# Table of Contents

- Calculation of multiplet frequency from cell-type mixing in `R`
  - Function to compute multiplet frequency
  - `R` function
  - Example when cell types mixed in equal proportion
  - Example when cell types are mixed unequally

# Calculation of multiplet frequency from cell-type mixing in `R`¶

Here we implement the simple function to calculate the multiplet frequency from single-cell RNA sequencing experiments where we have mixed cells of two types (e.g., human and mouse), and know the number of observed droplets that contain cells of each type.

## Function to compute multiplet frequency¶

The multiplet frequency is computed in terms of the following three experimental observables:

- the number of droplets that contain at least one cell of type 1, which we denote as $N\_1$
- the number of droplets that contain at least one of type 2, which we denote as $N\_2$
- the number of droplets containing cells of both type 1 and type 2, which we denote as $N\_{1,2}$

The multiplet frequency $M$ is
$$M = 1 - \frac{\left(\mu\_1 + \mu\_2\right)e^{-\mu\_1 - \mu\_2}}{1 - e^{-\mu\_1 - \mu\_2}}$$
where
$$\mu\_1 = -\ln\left(\frac{N - N\_1}{N}\right)$$
and
$$\mu\_2 = -\ln\left(\frac{N - N\_2}{N}\right),$$
and where
$$N = \frac{N\_1 N\_2}{N\_{12}}.$$

## `R` function¶

Here is the calculation implemented as `R` function:

In [1]:

```
#' Multiplet frequency from cell-type mixing experiment.
#'
#' @param n1 Number of droplets with at least one cell of type 1
#' @param n2 Number of droplets with at least one cell of type 2
#' @param n12 Number of droplets with cells of both types
#' @return The estimated multiplet frequency
multiplet_freq <- function(n1, n2, n12) {
  n <- n1 * n2 / n12
  mu1 <- -log((n - n1) / n)
  mu2 <- -log((n - n2) / n)
  mu <- mu1 + mu2
  return (1 - mu * exp(-mu) / (1 - exp(-mu)))
}
```

## Example when cell types mixed in equal proportion¶

First we demonstrate the calculations in the case when the cell types are mixed in equal proportions.

Let's create some hypothetical data.
We'll imagine that these data come from the 10X cellranger software analysis of a multi-species experiment that mixed mouse and human cells equally.
The current version (2.1.1) of the 10X cellranger pipeline for this type of study would return the *human Estimated Number of Cell Partitions*, the *mouse Estimated Number of Cell Partitions*, and the number of *GEMs with > 0 Cells* (this last quantity is also reported as the *Estimated Number of Cells*).
These statistics give the numbers of non-empty GEMs with cells of each type (GEMs is the term that 10X uses to refer to their droplets).

Here is a data frame of some hypothetical data from three different experiments, each with 4000 cells total but with different numbers of cross-celltype droplets:

In [2]:

```
df_equal <- data.frame(human_droplets=c(2005, 2050, 2500), 
           mouse_droplets=c(2005, 2050, 2500), 
           nonempty_droplets=c(4000, 4000, 4000), 
           row.names=c("experiment 1", "experiment 2", "experiment 3"))

df_equal
```

|  | human\_droplets | mouse\_droplets | nonempty\_droplets |
| --- | --- | --- | --- |
| experiment 1 | 2005 | 2005 | 4000 |
| experiment 2 | 2050 | 2050 | 4000 |
| experiment 3 | 2500 | 2500 | 4000 |

We calculate the number of droplets with cells of **both** types (human and mouse) simply as the sum of the human and mouse droplets minus the total number of non-empty droplets, since these cross-celltype droplets are double counted in the tally of human and mouse droplets.
As is apparent after this calculation, in this hypothetical example the cross-celltype droplets represent 0.25%, 2.5%, and 25% of the total non-empty droplets in the three examples.

Note that we need to import `dplyr` to run the next cell:

In [3]:

```
library(dplyr)

df_equal <- df_equal %>%
  mutate(human_and_mouse_droplets=
    human_droplets + mouse_droplets - nonempty_droplets)

df_equal
```

```
Attaching package: ‘dplyr’

The following objects are masked from ‘package:stats’:

    filter, lag

The following objects are masked from ‘package:base’:

    intersect, setdiff, setequal, union
```

| human\_droplets | mouse\_droplets | nonempty\_droplets | human\_and\_mouse\_droplets |
| --- | --- | --- | --- |
| 2005 | 2005 | 4000 | 10 |
| 2050 | 2050 | 4000 | 100 |
| 2500 | 2500 | 4000 | 1000 |

We now calculate the multiplet frequency in two ways.
The first way is to precisely calculate the multiplet frequency using the exact Poisson derivation as implemented in the `multiplet_freq` function above.

The second way is to do the simple calculation that has commonly been used in paper that do equal-proportion mixing.
This method is to simply estimate the multiplet frequency as twice the frequency of cross-cell-type droplets among all non-empty droplets (that is, as $\frac{2 \times N\_{1,2}}{N\_1 + N\_2 + N\_{12}}$).

In [4]:

```
df_equal <- df_equal %>%
  mutate(
    multiplet_freq=multiplet_freq(
      human_droplets, mouse_droplets, human_and_mouse_droplets),
    twice_cross_celltype_freq=
      2 * human_and_mouse_droplets / nonempty_droplets
    )

df_equal %>% format(digits=2)
```

| human\_droplets | mouse\_droplets | nonempty\_droplets | human\_and\_mouse\_droplets | multiplet\_freq | twice\_cross\_celltype\_freq |
| --- | --- | --- | --- | --- | --- |
| 2005 | 2005 | 4000 | 10 | 0.005 | 0.005 |
| 2050 | 2050 | 4000 | 100 | 0.049 | 0.050 |
| 2500 | 2500 | 4000 | 1000 | 0.425 | 0.500 |

As can be seen above, the two methods give virtually identical results as long as the number of cross-celltype droplets is small relative to the total number of droplets.
The reason that the two methods aren't identical as simply calculating the multiplet frequency as twice the cross-celltype frequency neglects to account for droplets that have more than two cells.
However, this difference only becomes appreciable when the multiplet frequency is high.
So for the examples above, we can see that it only really matters in the case when the true multiplet frequency is $\approx$0.425; in that case, simply taking twice the cross-celltype frequency slightly overestimates the true multiplet frequency

## Example when cell types are mixed unequally¶

Now we repeat the example above, but for experiments where the cell types are mixed unequally.

Below we give some example calculations for various numbers.
An interesting (and initially non-intuitve aspect) of the results is that when the cells are mixed highly unequally and multiplets are common, the multiplet frequency is actually substantially **less** than the fraction of droplets with the rare cell type that are multiplets.
The reason is that multiplets are more likely than singlets to have a cell of the rarer type, and become progressively more likely to have a cell of the rare type as the number of cells in the multiplet increases.

In [5]:

```
data.frame(
    human_droplets=c(2050, 3050, 3550, 3850, 3950),
    mouse_droplets=c(2050, 1050, 550, 250, 150),
    nonempty_droplets=c(4000, 4000, 4000, 4000, 4000)
    ) %>%
  mutate(
    human_and_mouse_droplets=
      human_droplets + mouse_droplets - nonempty_droplets,
    multiplet_freq=multiplet_freq(
      human_droplets, mouse_droplets, human_and_mouse_droplets)
    ) %>%
  format(digits=2)
```

| human\_droplets | mouse\_droplets | nonempty\_droplets | human\_and\_mouse\_droplets | multiplet\_freq |
| --- | --- | --- | --- | --- |
| 2050 | 2050 | 4000 | 100 | 0.049 |
| 3050 | 1050 | 4000 | 100 | 0.065 |
| 3550 | 550 | 4000 | 100 | 0.110 |
| 3850 | 250 | 4000 | 100 | 0.245 |
| 3950 | 150 | 4000 | 100 | 0.459 |
